# Hospitalisation and critical care for pneumonia among children aged 5–9 years in Bangladesh: a 10-year retrospective analysis

**DOI:** 10.7189/jogh.15.04326

**Published:** 2025-12-05

**Authors:** Haimanti Saha, Farzana Afroze, Lubaba Shahrin, Monira Sarmin, Rukaeya Amin, Mosharrat Tabassum, Nafisa Mariam, Al-Afroza Sultana, Shamsun Nahar Shaima, Md Zahidul Islam, Abu Sadat Mohammad Sayeem Bin Shahid, Tahmeed Ahmed, Mohammod Jobayer Chisti

**Affiliations:** 1 Clinical and Diagnostic Services, International Centre for Diarrhoeal Disease Research, Bangladesh, Dhaka, Bangladesh.; 2 Nutrition Research Division, International Centre for Diarrhoeal Disease Research, Bangladesh, Dhaka, Bangladesh; 3 Department of Nutritional Sciences, School of Graduate Studies, University of Toronto, Toronto, Ontario, Canada

## Abstract

**Background:**

Most medical research on pneumonia in children focuses on those <5 years, leaving a gap in understanding pneumonia in children aged 5–9. We aimed to identify the characteristics of children from this age group who had pneumonia and required hospital care, including critical care service.

**Methods:**

In this retrospective chart analysis, we examined clinical, demographic, and laboratory characteristics of children aged 5–9 years with clinical and radiologic pneumonia admitted to Dhaka Hospital, International Centre for Diarrhoeal Disease Research, Bangladesh, from 2011 to 2020. We categorised the children into two groups: those who required critical care (admitted to the intensive care unit (ICU)) and those who did not. We compared the two groups to identify factors independently associated with the need for critical care using a log binomial regression model.

**Results:**

Among a total of 154 children who fulfilled the enrolment criteria, 34 were admitted to the ICU requiring critical care, and 120 children were treated in the inpatient ward, as they did not require any critical care. The median age of the children requiring critical care was 69 (interquartile range (IQR) = 60–81) months, compared to 72 (IQR = 62–84) months for those who didn`t require critical care (*P* = 0.259). Using a log binomial regression model we found hypoxemia (odds ratio (OR) = 10.1; 95% confidence interval (CI) = 1.42–71.92, *P* = 0.021), convulsion (OR = 281.37; 95% CI = 12.99–6091.72, *P* < 0.001], sepsis (OR = 27.69; 95% CI = 3.33–230.39, *P* = 0.002), hypokalaemia (OR = 10.37; 95% CI = 1.40–76.96, *P* = 0.022) were the independently associated with critical care service among children aged five to nine with pneumonia.

**Conclusions:**

Our results suggest that early recognition and prompt treatment of hypoxemia, convulsions, sepsis, and hypokalaemia may significantly reduce the need for critical care and possibly avert fatal consequences in children with pneumonia, aged 5–9, especially in resource-limited settings.

In 2017, global child mortality statistics revealed that nearly one million children aged 5–14 years lost their lives, with 98% of these deaths occurring in low- and middle-income countries [1,2]. Despite historical focus on reducing child mortality in children <5 years, older children and adolescents have been largely overlooked in terms of morbidity and mortality. Given that pneumonia is a leading cause of death, it is essential to assess all age groups, including those older than five years, for effective planning and research [3-5]. Global Burden of Disease estimates suggest that pneumonia accounts for approximately 7% of deaths among children aged 5–9 [6]. Although children aged 5–9 years are generally considered at lower risk for pneumonia-related deaths, certain conditions, such as chronic illnesses or physical debility, can elevate their risk. In addition, co-existing conditions such as malaria, asthma, neurological disorders, severe malnutrition, and HIV are frequently observed in this age group [7]. The evidence on risk factors for death in children older than five years with community-acquired pneumonia remains extremely limited, and the aetiology or risk association of severe disease has yet to be explored. The World Health Organization (WHO) criteria for classification of severity for children <5 years do not seem to be an effective tool for risk assessment in this older age group, indicating the urgent need for evidence-based clinical strategies [4]. Currently, there is limited evidence on risk factors for death in children older than five years with community-acquired pneumonia, highlighting the urgent need for age-specific clinical strategies for this population. Furthermore, there is a lack of studies evaluating the incidence of severe pneumonia and intensive care unit (ICU) admission rates for this age group. The Dhaka Hospital, International Centre for Diarrhoeal Disease Research, Bangladesh (icddr,b), frequently treats patients of all ages with pneumonia. While there have been numerous studies on pneumonia among children under the age of five, there is a noticeable number of pneumonic children aged 5–9, often requiring respiratory support with critical care service, but lacking published evidence. This highlights the importance of further understanding and addressing respiratory issues in this age group. Thus, we aimed to identify risk factors for critical care services in children aged 5–9 years with pneumonia in Bangladesh. This is crucial because no such studies are currently available, and the findings could significantly contribute to the development of effective interventions for this age group, especially in low- and middle-income countries.

## METHODOS

### Study design

In this retrospective case-control study, we used de-identified data from January 2011 to December 2020 on children aged 5–9 years in the icddr,b electronic medical record system, known as Sheba.

### Study settings

At the Dhaka Hospital of icddr,b, over 200 000 patients, spanning all ages and genders, receive comprehensive care and treatment for diarrhoea and its related complications annually [8]. The hospital primarily admits patients with diarrhoea, malnutrition, and acute respiratory infections. Notably, children exhibiting symptoms, such as respiratory distress, cyanosis, apnea, hypothermia, sepsis, shock, impaired consciousness, convulsions, severe pneumonia with hypoxemia, or respiratory failure are promptly transferred to the hospital's ICU for immediate and specialised medical attention. It is important to note that most of the hospital's patients are from low-income families, predominantly residing in urban and peri-urban areas of Dhaka. This contextual understanding underscores the hospital's crucial role in serving vulnerable populations and addressing critical health needs within these communities.

### Operational definition

Pneumonia is defined as a patient presenting with cough and/or respiratory difficulty, with evidence of end-point consolidation or other (non-endpoint) infiltrates on chest radiograph, according to the WHO radiological classification of pneumonia [9]. A qualified practitioner radiologist independently confirmed the diagnosis. Acute kidney injury (AKI) is defined as elevated serum creatinine (>1.5 times the reference value). The normal reference value for children is 21–65 μmol/L, so those with creatinine >97.5 μmol/L are considered to have AKI [10]. Furthermore, critical care service refers to the requirement of ICU care. Lastly, respiratory failure is determined by an oxygen saturation/fraction of inspired oxygen (SpO_2_/FiO_2_) of <315 [11].

### Inclusion criteria

We included individuals who met criteria for a history of fever, cough with or without tachypnea, and crepitus, and radiological evidence of pneumonia. Therefore, pneumonia was confirmed based on radiological evidence. These patients were admitted to the ICU or the general ward based on the severity of their condition. We compared the characteristics of children aged 5–9 years who required critical care services with those who did not.

### Patient management and measurements

The approach to treating children with severe pneumonia and hypoxemia at the ICU of Dhaka Hospital in icddr,b depends on whether they show signs of respiratory failure upon arrival. Oxygen therapy and respiratory support approaches, including non-invasive ventilation and mechanical ventilation, are applied based on clinical assessment and severity. The study children were given antibiotics and the WHO standard oxygen therapy for hypoxemia. They also received routine supportive care in accordance with the hospital’s standard guidelines. We used case report forms to collect data on major clinical parameters, including demographics (age, gender), nutritional status, dehydration, severe sepsis, mental status, convulsion at admission, respiratory rate, temperature, and laboratory parameters. We retrospectively retrieved the data from a computer-based patient management system. Upon admission to the Dhaka Hospital, each patient is assigned a unique identifying number, against which all their data are recorded. This includes history, clinical examination findings, laboratory reports, treatment provided, dietary management, daily follow-up, and clinical outcomes.

### Data analysis

We used STATA, version 15.0 (StataCorp LLC, College Station, Texas, USA) for all analyses. We presented categorical variables as numbers (percentages), measured their associations using the χ^2^ or Fisher's exact test, and reported the strength of association using the odds ratio (OR). We presented continuous variables as means (standard deviations) or medians (interquartile ranges (IQRs)), and measured their associations using the *t*-test or Mann-Whitney test. We assessed the normality of continuous variables using the Shapiro-Wilk test. Finally, we assessed the factors associated with the need for critical care services using a log-binomial regression model. For the regression analysis, we included variables that were significant in the bivariate analysis at the 5% level and those that were clinically important for ICU admission.

## RESULTS

**Figure 1:**
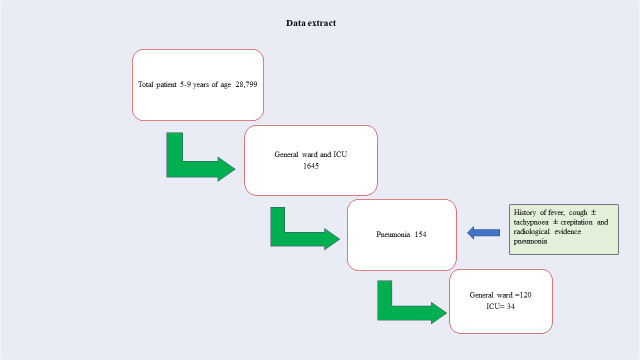
Data extract. ICU – intensive care unit.

Between January 2011 and December 2020, 28 799 children aged 5–9 required hospitalisation. Among them, 1645 patients were admitted to inpatient facilities, including the general ward and the ICU. We included 154 patients who met the criteria for pneumonia. Among the 154 participants, 120 (78%) were admitted to the general ward, and 34 (22%) required critical care service (**Figure 1**). All patients in both groups were discharged after full clinical recovery, except three who were referred to higher facilities for their concomitant other illnesses. The median age for critical care service was 69 months (IQR = 60–81), while it was 72 months (IQR = 62–84) for general ward admission (**Table 1**). There was a slightly higher proportion of males than females in both groups; however, this difference was not statistically significant (OR = 1.07; 95% CI = 0.50–2.31, *P* = 0.859). Fever was not associated with critical care service (OR = 2.11; 95% CI = 0.46–9.79, *P* = 0.339). However, cough (OR = 0.30; 95% CI = 0.11–0.82, *P* = 0.020), hypoxemia (OR = 5.14; 95% CI = 1.89–14.01, *P* = 0.001), altered mental status (OR = 8.68; 95% CI = 3.40–22.16, *P* < 0.001), and convulsions (OR = 38.08; 95% CI = 4.56–318.2, *P* = 0.001) were identified as significant clinical features for critical care service. In addition, diarrhoea (OR = 6.63; 95% CI = 2.40–18.28, *P* < 0.001), vomiting (OR = 3.16; 95% CI = 1.40–7.11, *P* = 0.005), abdominal distention (OR = 7.87; 95% CI = 1.38–45.00, *P* = 0.020), and sepsis (OR = 18.65; 95% CI = 4.82–72.14, *P* < 0.001) were found to be significantly associated with the critical care service group.

**Table 1:** Sociodemographic and clinical characteristics of children by ICU admission*

	Total pneumonia children (n = 154)	Required ICU admission (n = 34)	Non-required ICU-admission (n = 120)	OR (95% CI)	*P*-value
**Demographic characters**					
Age in months, MD (IQR)	72.0 (60.0–84.0)	69.0 (60.0–81.0)	72.0 (62.0–84.0)	0.99 (0.96–1.02)	0.379
Male sex	84 (55)	19 (56)	65 (54)	1.07 (0.50–2.31)	0.859
**Major clinical features**					
Fever	138 (90)	32 (94)	106 (88)	2.11 (0.46–9.79)	0.339
Cough	136 (88)	26 (76)	110 (92)	0.30 (0.11–0.82)	0.020
Hypoxemia	19 (12)	10 (29)	9 (7)	5.14 (1.89–14.01)	0.001
Abnormal mental status	129 (84)	19 (56)	110 (92)	8.68 (3.40–22.16)	<0.001
Convulsion	9 (6)	8 (24)	1 (1)	38.08 (4.56–318.22)	0.001
**Diarrhoea-related parameters**					
Diarrhoea	85 (55)	29 (85)	56 (47)	6.63 (2.40–18.28)	<0.001
AWD	70 (45)	23 (68)	47 (39)	3.25 (1.45–7.28)	0.004
ID	12 (08)	5 (15)	7 (6)	2.78 (0.82–9.41)	0.100
Dehydration	132 (86)	24 (71)	108 (90)	3.75 (1.45–9.68)	0.006
Vomiting	39 (25)	15 (44)	24 (20)	3.16(1.40–7.11)	0.005
Abdominal distension	6 (4)	4 (12)	2 (2)	7.87 (1.38–45.00)	0.020
**Comorbid presence of other clinical features**					
Anaemia	94 (61)	18 (53)	76 (63)	1.54 (0.71–3.31)	0.274
Asthma	31 (20)	3 (9)	28 (23)	0.32 (0.09–1.12)	0.074
CHD	6 (4)	3 (9)	3 (3)	3.77 (0.73–19.62)	0.114
Pulmonary TB	6 (4)	3 (9)	3 (3)	3.77 (0.73–19.62)	0.114
Sepsis	14 (9)	11 (32)	3 (3)	18.65 (4.82–72.14)	<0.001
UTI	15 (10)	4 (12)	11 (9)	1.32 (0.39–4.45)	0.653
HAI	2 (1)	1 (3)	1 (1)	3.61 (0.22–59.21)	0.369
**Vital signs, MD (IQR)**					
Temperature, °C	38.0 (37.0–39.0)	38.5 (37.8–39.0)	37.9 (37.0–39.0)	1.56 (1.10–2.20)	0.011
Respiratory rate, breaths/min	40.0 (34.0–48.0)	44.0 (38.0–48.0)	38.0 (32.0–46.0)	1.03 (0.99–1.07)	0.099
S_P_O_2_	95.0 (92.0–98.0)	96.5 (88.5–98.0)	96.0 (93.0–98.0)	0.64 (0.55–0.75)	<0.001
**Laboratory parameters**					
Hyponatremia	51 (33)	26 (76)	25 (21)	12.35 (4.99–30.58)	<0.001
Hypokalaemia	30 (19)	15 (44)	15 (13)	5.53 (2.32–13.15)	<0.001
Acidosis	27 (18)	15 (44)	12 (10)	7.11 (2.88–17.51)	<0.001
Alkalosis	10 (6)	5 (15)	5 (4)	3.97 (1.08–14.62)	0.039
AKI	7 (5)	6 (18)	1 (1)	25.5 (2.95–220.38)	0.003
Hypocalcaemia	15 (10)	13 (38)	2 (2)	36.52 (7.68–173.70)	<0.001

AKI – acute kidney injury, AWD – acute watery diarrhoea, CHD – congenital heart disease, CI – confidence interval, HAI – hospital-associated infection, ICU – intensive care unit, ID – invasive diarrhoea, IQR – interquartile range, MD – median, OR – odds ratio, TB – tuberculosis, UTI – urinary tract infection

*Values presented as n (%) unless specified otherwise.

**Figure 2:**
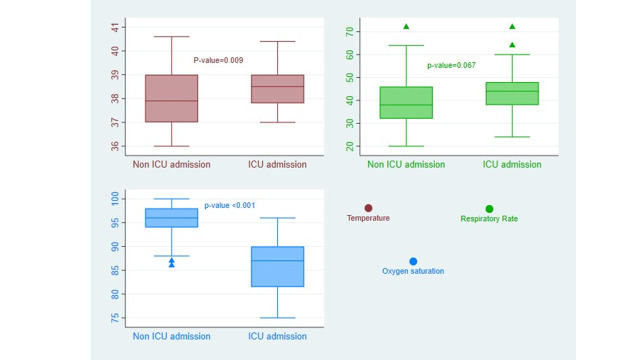
Comparison of admission vital signs between those who received critical care service and those who did not. **Panel A.** Temperature. **Panel B.** Respiratory rate. **Panel C.** Oxygen saturation. ICU – intensive care unit.

A considerable number of children had additional health issues, such as anaemia, asthma, congenital heart disease, urinary tract infection, pulmonary tuberculosis, and sepsis. However, only sepsis showed a significant association with critical care service in the preliminary bivariate analysis. Among the laboratory findings, hyponatremia, hypokalaemia, hypocalcaemia, acidosis, alkalosis, and AKI with a creatinine increase of more than 1.5 times were significantly linked to critical care service in the initial analysis. Upon admission, the median temperature and respiratory rate were elevated, and oxygen saturation was low in the group that received critical care services (**Figure 2**, Panels A–C).

Through regression analysis using a log-linear binomial model, we found that hypoxemia (OR = 10.10; 95% CI = 1.42–71.92, *P* = 0.021), convulsion (OR = 281.37; 95% CI = 12.99–6091.72, *P* < 0.001), congenital heart disease (OR = 7.41; 95% CI = 0.84–65.02, *P* = 0.071), sepsis (OR = 27.69; 95% CI = 3.33–230.39, *P* = 0.002), and hypokalaemia (OR = 10.37; 95% CI = 1.40–76.96, *P* = 0.022) are the independent associated factors for the need of critical care services among children aged 5–9 years who have pneumonia (**Table 2**).

**Table 2:** Associated factors for ICU admission

	OR (95% CI)	*P*-value
**Older age group (>72 months)**	1.16 (0.32–4.20)	0.823
**Male sex**	0.37 (0.09–1.43)	0.149
**ID**	0.42 (0.05–3.84)	0.443
**Cough**	0.23 (0.04–1.40)	0.112
**Vomiting**	1.37 (0.31–5.95)	0.677
**Abdominal distension**	5.48 (0.34–88.28)	0.230
**Hypoxemia**	10.10 (1.42–71.92)	0.021
**Asthma**	1.56 (0.20–11.94)	0.666
**CHD**	7.41 (0.84–65.02)	0.071
**Convulsion**	281.37 (12.99–6091.72)	<0.001
**Hyponatremia**	3.42 (0.49–23.73)	0.214
**Hypokalaemia**	10.37 (1.40–76.96)	0.022
**Acidosis**	0.97 (0.18–5.28)	0.968
**Alkalosis**	3.52 (0.45–27.32)	0.228
**AKI**	6.57 (0.38–113.97)	0.196
**Sepsis**	27.69 (3.33–230.39)	0.002

AKI – acute kidney injury, CHD – congenital heart disease, CI – confidence interval, ICU – intensive care unit, ID – invasive diarrhoea, OR – odds ratio

## DISCUSSION

We provide a detailed description of the characteristics of children aged 5–9 with pneumonia who required critical care services compared to those who did not.

Among all children aged 5–9 years hospitalised for pneumonia, we observed that 22% children required critical care services. This rate is higher than that of the paediatric age group (13.8%) and the adult population (19%) in previous studies [12,13]. However, data on critical care services for the 5–9 age group were not reported in most studies [12,14]. Once the patient is admitted to the ICU, several prognostic factors known from previous studies are typically associated with increased mortality; however, in our study, no deaths occurred.

We observed that the odds of having hypoxemia were ten times higher in the group of ICU patients compared to the non-ICU patients’ group. Several previous studies also revealed a similar proportion of children with pneumonia having hypoxemia requiring critical care services [15-17].

Another important observation was the convulsion, which was identified as an independent associated factor for requiring critical care services. In children under five, convulsions are considered a sign of severe pneumonia and indicate the need for essential care services. Although convulsions have been identified as an independent associated factor for mortality in children under five years of age in previous studies [18], we did not observe this association in children aged 5–9 years.

A borderline association between congenital heart disease and the need for critical care service was also reported in earlier studies [19]. Moreover, the high prevalence of congenital heart disease in the 5–9-year age group supports our observation [20]. This is mainly because the presence of congenital heart disease may contribute to repeated respiratory tract infections, which in turn increases the risk of ICU admission and subsequent requirement of critical care service.

We observed that the odds of having asthma were 1.5 times higher among ICU patients. However, this association was not independently significant for ICU admission in our final regression analysis. Nonetheless, the coexistence of asthma and pneumonia aligns with findings from earlier studies [18]. Pneumonia is recognised as a contributing factor to acute asthma exacerbation in this age group [21]. We identified that the odds of having sepsis were 27 times higher among ICU patients in children aged 5–9. This aligns with findings from other studies, which have identified pneumonia-related sepsis as a major contributing factor for ICU admission, in both the under-five and adult populations [22-24].

Electrolyte imbalance is common in pneumonia, especially hyponatremia and hypokalaemia. This can complicate the outcome of pneumonia and prolong hospital stays, as indicated by several studies [25,26]. We also discovered a substantial number of children presented with hyponatremia and hypokalaemia. In our final model, the odds of hypokalaemia were ten times higher among ICU admissions and were independent predictors of receiving critical care services.

The primary limitation of our study is its retrospective design, which may have prevented us from exploring several important variables. Additionally, the small sample size may have reduced the statistical power of our analyses. Lastly, we lacked information on the outcomes of patients referred to higher-level facilities.

## CONCLUSIONS

We found that children aged 5–9 with pneumonia presenting with hypoxemia, convulsions, sepsis, or hypokalaemia are at higher risk of requiring critical care services. Early identification of these simple clinical signs and timely treatment may help clinicians reduce ICU admissions and improve outcomes, particularly in resource-limited settings.
